# Extracts from *Eryngium heterophyllum* Engelm. [Apiaceae] enhance paclitaxel cytotoxicity in A549 lung cancer cells

**DOI:** 10.3389/fphar.2026.1854397

**Published:** 2026-09-03

**Authors:** Blanca Elizabeth Hernandez Solano, Joel Daniel Castañeda-Espinoza, Enrique Salas-Vidal, Araceli Guerrero-Alonso, Janette Furuzawa-Carballeda, Carlos Mojica Cardoso, Abraham Noé Anzurez Jiménez, Jessica Nayelli Sánchez-Carranza, Laura Alvarez

**Affiliations:** 1 Facultad de Farmacia, Universidad Autónoma del Estado de Morelos, Cuernavaca, Morelos, Mexico; 2 Centro de Desarrollo de Productos Bióticos, Instituto Politécnico Nacional, Yautepec, Morelos, Mexico; 3 Departamento de Genética del Desarrollo y Fisiología Molecular, Instituto de Biotecnología, Universidad Nacional Autónoma de México, Cuernavaca, Morelos, Mexico; 4 Centro de Investigaciones Químicas-IICBA, Universidad Autónoma del Estado de Morelos, Cuernavaca, Morelos, Mexico; 5 Departamento de Cirugía Experimental, Instituto Nacional de Ciencias Médicas y Nutrición Salvador Zubirán, Ciudad de México, Mexico; 6 Escuela de Medicina, Universidad Panamericana, Ciudad de México, Mexico; 7 Laboratorio de Patología, Hospital del Niño Morelense, Emiliano Zapata, Morelos, Mexico

**Keywords:** chemosensitization, combination therapy, *Eryngium heterophyllum*, lung cancer, natural extracts, paclitaxel

## Abstract

**Introduction:**

Chemotherapy resistance is a critical bottleneck in lung cancer treatment, significantly limiting the clinical efficacy of drugs like paclitaxel. This study investigated the cytotoxic and chemosensitizing potential of hexane, acetone, and hydroalcoholic extracts from *Eryngium heterophyllum* Engelm. [Apiaceae] to enhance therapeutic response in A549 lung cancer cells.

**Methods:**

Extracts were obtained by maceration and their antiproliferative activity was determined using crystal violet assays. Synergistic interactions between extracts and paclitaxel were quantified using the Chou–Talalay combination index. Mechanistic studies included DAPI staining for nuclear fragmentation, immunofluorescence for P-glycoprotein expression, and flow cytometry to assess functional doxorubicin retention. Chemical profiling was performed via Gas Chromatography-Mass Spectrometry.

**Results:**

The hexane extract exhibited the highest intrinsic cytotoxicity (IC_50_ = 25.1 ± 1.1 μg/mL). Notably, combining sub-cytotoxic concentrations of the extracts with paclitaxel reduced the drug’s IC_50_ by 10 to 14-fold, with the acetone extract showing the strongest synergism. These combinations induced significant apoptotic nuclear fragmentation. Furthermore, all extracts increased intracellular doxorubicin retention to levels comparable to the reference inhibitor verapamil, suggesting potent inhibition of P-gp mediated efflux.

**Discussion:**

These findings demonstrate that *E. heterophyllum* extracts exhibit a dual biological effect by providing direct antiproliferative activity while also enhancing the response of A549 cells to paclitaxel. The observed effects may be associated with modulation of drug transport-related mechanisms, including increased intracellular doxorubicin retention and changes in P-gp associated activity. This species represents a promising source of plant-derived metabolites as complementary agents for improving chemotherapy response in lung cancer cells.

## 1 Introduction

Lung cancer remains the leading cause of cancer-related mortality worldwide, with the latest global data estimating approximately 2.48 million new cases and 1.8 million deaths in 2022, representing nearly 20% of all cancer deaths globally. These statistics highlight the ongoing public health burden of lung cancer and the need for improved therapeutic strategies to enhance treatment efficacy and overcome limitations of current chemotherapy regimens (Zhou et al., 2024; Bray et al., 2024; Thai et al., 2021).

Despite significant advances in targeted therapies and immunotherapy, chemotherapy continues to be a cornerstone in the treatment of advanced NSCLC. Although targeted therapies and immune checkpoint inhibitors have transformed the treatment landscape of non-small cell lung cancer (NSCLC), conventional chemotherapy remains a key option, especially for patients who are ineligible for or progress after these treatments. Paclitaxel (PTX), a taxane-based microtubule-stabilizing agent, is widely used in clinical regimens for advanced NSCLC, often in combination with platinum (Ramalingam and Belani, 2004; Rosell et al., 2002; Tan et al., 2023).

However, the clinical utility of PTX is frequently compromised by the development of drug resistance. Recent studies have documented diverse mechanisms of PTX resistance in NSCLC, including drug efflux transporters, alterations in microtubule dynamics, and regulatory pathways that limit cell death responses. Such mechanisms contribute to treatment failure and underscore the need for strategies that enhance PTX cytotoxicity (Orr et al., 2003; Alalawy, 2024).

Strategies aimed at enhancing the cytotoxic efficacy of conventional chemotherapeutic agents have gained increasing attention. In particular, the use of natural products as adjuvant agents represents a promising approach to improve cancer treatment outcomes. Indeed, different extracts of plants and phytochemicals have been shown to enhance the antitumor activity of conventional chemotherapeutic agents by modulating cellular processes related to proliferation, cell death, oxidative stress, and drug transport mechanisms (Newman and Cragg, 2020). Such multitarget effects support their potential role in combination strategies which are consistent with current pharmacological innovation approaches that seek to improve therapeutic outcomes through the integration of bioactive metabolites derived from traditional medicinal sources (Alalawy, 2024; Cui et al., 2020; Wiese and Zheng, 2006; Atanasov et al., 2021).

The genus *Eryngium* [Apiaceae]comprises numerous species traditionally used botanical drugs and is recognized as a source of bioactive secondary metabolites, including phenolic metabolites and flavonoids. Several *Eryngium* species have been reported to exhibit antiproliferative and cytotoxic activities in different cancer models (Cárdenas-Valdovinos et al., 2023).

*Eryngium heterophyllum*, a species widely distributed in México, particularly in central and western regions, has been traditionally used for multiple therapeutic purposes. In Mexican traditional medicine, it has been employed for the management of metabolic disorders, including hypercholesterolemia, as well as for its anti-inflammatory, analgesic, and diuretic properties. Additionally, it has been used in the treatment of gastrointestinal disturbances, respiratory conditions, and as a general tonic. Phytochemical studies have revealed that this species contains diverse bioactive metabolites, such as flavonoids, phenolic acids, and other secondary metabolites associated with antioxidants, hypolipidemic, and cytotoxic activities. Previous reports have demonstrated that extracts of *E. heterophyllum* exhibit significant biological effects, including antiproliferative activity in cancer cell lines, supporting its potential as a source of anticancer agents (Cárdenas-Valdovinos et al., 2023; Villa-Santiago et al., 2025; Wang et al., 2012).

Phytochemical studies of *E. heterophyllum* have reported the presence of metabolites such as rutin, ferulic acid, caffeic acid, gallic acid, β-sitosterol, falcarindiol, stigmasterol, and D-mannitol in different plant parts and extracts (Villa-Santiago et al., 2025; Galván-Ciprés et al., 2026). However, the phytochemical composition of this species appears to vary according to extraction conditions and solvent polarity, and the metabolites potentially associated with chemosensitizing related effects remain poorly characterized.

Previous studies, including our earlier work on *E. heterophyllum*, demonstrated the cytotoxic and selective effects of different extracts against cancer cells, supporting the pharmacological potential of this species (Castañeda-Espinoza et al., 2026). However, those studies were mainly focused on the intrinsic cytotoxic activity and phytochemical characterization of the extracts. Based on these findings, the present study was designed to investigate whether *E. heterophyllum* extracts could enhance the response of A549 lung cancer cells to PTX through potential chemosensitizing effects associated with modulation of drug transport-related mechanisms. In addition to evaluating cytotoxicity and intracellular doxorubicin retention, this study provides preliminary evidence supporting the potential use of *E. heterophyllum* as a source of natural adjuvant agents in chemotherapy. Furthermore, the present work establishes a foundation for future studies aimed at identifying the bioactive metabolites involved and evaluating their activity in resistant lung cancer models.

## 2 Materials and methods

### 2.1 Drugs, chemicals, and instruments

Solvents used for extraction, including *n*-hexane (98.99% purity), acetone (98.8% purity), and methanol (99.9% purity), as well as distilled water (high purity grade), were purchased from J.T. Baker (Phillipsburg, NJ, USA). Dulbecco’s Modified Eagle’s Medium (DMEM), fetal bovine serum (FBS), L-glutamine, streptomycin, penicillin, and Whatman No. 6 filter paper were obtained from Invitrogen, Thermo Fisher Scientific, Inc. (Waltham, MA, USA). Verapamil, doxorubicin, PTX, and crystal violet (≥90% purity, CAS No. 548-62-9) were purchased from Sigma-Aldrich (St. Louis, MO, USA).

Plant material processing was performed using an electric microfine grinder mill (Basic Microfine Motor MF 10; IKA®, Wilmington, NC, USA). Evaporation processes were carried out using a R-114 rotary evaporator (Büchi Labortechnik AG, Flawil, Switzerland). Analytical weighing was performed using an Ohaus PR124 analytical balance (Ohaus Corporation, Parsippany, NJ, USA). Cell culture experiments were conducted using a CO_2_ incubator (UN-5700, NuAire, Plymouth, MN, USA). Cytotoxicity and fluorescence analyses were performed using a Promega automated ELISA reader (Madison, WI, USA) and an Olympus BX61 fluorescence microscope (Olympus, PA, USA), respectively. Flow cytometric analysis was carried out using a BD FACSLyric™ flow cytometer (Becton Dickinson, San Jose, CA, USA).

For immunofluorescence assays, the primary antibody anti-P-gp mouse (1:100, sc-55510, Santa Cruz Biotechnology, Dallas, TX, USA) and the secondary antibody anti-mouse Alexa Fluor 647 (1:1000, A-21235, Molecular Probes, Thermo Fisher Scientific, Inc., Waltham, MA, USA) were used. DAPI (1:2500, D9542, Sigma-Aldrich, St. Louis, MO, USA) was used for nuclear staining, and samples were mounted using Aqueous Mounting Medium #27290 (Cell Signaling Technology). Samples were visualized using an Olympus FluoView FV1000 confocal microscope system coupled to an inverted IX81 Olympus microscope (Olympus, Tokyo, Japan) equipped with a UPlanSApo ×40 objective (numerical aperture 0.75).

### 2.2 Plant material

Leaves and stems of wild *E. heterophyllum* Engelm. [Apiaceae] plants were collected in the municipality of Ixtapan de la Sal, State of México (18°53′25″N, 99°40′24″W) State of México in February 2024. The taxonomic identification was done by Biol. Gabriel Flores Franco at the HUMO Herbarium of the Centre for Research in Biodiversity and Conservation of the Universidad Autónoma del Estado de Morelos with voucher number 29010. For its taxonomic determination, the identification keys included in the Contribution to the knowledge of the genus Eryngium [Apiaceae] in the state of Michoacán, Mexico (García-Ruiz, 2013) and in the treatment of the Apiaceae family in the Flora of the Tehuacán-Cuicatlán Valley (López-Ferrari, 2020) were used. The taxonomic identity was corroborated using the Catalog of Native Vascular Plants of Mexico (Villaseñor, 2016).

### 2.3 Obtaining hexane, acetone and hydroalcoholic extracts from *Eryngium heterophyllum*

The plant material was left to dry at room temperature and then crushed. Maceration extraction was performed at room temperature (25 °C ± 3 °C) following the methodology described by Castañeda-Espinoza et al. (2026). For this purpose, 4.0 kg of plant material was extracted consecutively, first with n-hexane, followed by acetone, and finally with a methanol: water mixture in a ratio of 80:20 *(v/v)* in triplicate and for 72 h per extraction step.

Each extract was filtered using Whatman No. 6 filter paper, and the solvent was removed under reduced pressure by rotary evaporation. The concentration process was conducted at 40 °C and 300 mbar for the hexanic and acetonic extracts, and at 60 °C and 150 mbar for the hydroalcoholic extract. The yield percentage for the hexane extract was 1% (40 g), for the acetone extract 2% (80 g), and for the hydroalcoholic extract 5.5% (330 g).

### 2.4 Cell culture

The A549 non-small cell lung adenocarcinoma and HFF-1 human foreskin fibroblasts cell lines were acquired from the American Type Culture Collection (ATCC; Manassas, VA, USA), cultured in DMEM medium and supplemented with 10% fetal bovine serum (FBS), 2 mM glutamine, penicillin (100 U/mL) and streptomycin (0.1 mg mL-1) at 37 °C in an atmosphere containing 5% CO_2_.

### 2.5 Preparation of stock solutions of extracts

The hexane, acetone, and hydroalcoholic extracts were named according to the solvents used during the extraction procedure. After extraction, the solvents were completely removed under reduced pressure, yielding the extracts as dried residues. Prior to biological evaluation, the extracts were diluted in dimethyl sulfoxide (DMSO).

DMSO was used as the sole vehicle in all experiments, including cytotoxicity, chemosensitization, and doxorubicin retention assays. The final DMSO concentration never exceeded 0.1% (v/v), and control groups with an equivalent vehicle were included in all assays. Under these conditions, DMSO did not affect cell viability.

### 2.6 Cytotoxicity assay

5,000 cells per well were cultured in a 96-well plate, treated with the extracts for 48 h at concentrations of 200, 100, 50, 25, and 12.5 μg/mL to obtain a concentration-response curve, and incubated at 37 °C in a 5% CO_2_ atmosphere. Subsequently, the number of viable cells was determined using crystal violet (Sigma-Aldrich) (Feoktistova et al., 2016). Cell viability was determined by absorbance at 570 nm using an automatic ELISA reader. The experiments were performed in triplicate in three independent experiments. The data were analyzed using the GraphPad Prism 8.0 statistical program, and the IC_50_, IC_25_, IC_15_ and IC_10_ values for the treatments in combination with PTX were determined by nonlinear regression analysis.

### 2.7 Sensitivity to PTX in A549 cells

Due to the greater intrinsic cytotoxic activity of the hexane and acetone extracts, concentrations corresponding to their IC_10_ values were selected for the combined assays to minimize the direct cytotoxic contribution of the extracts during concomitant PTX treatment. Conversely, the hydroalcoholic extract showed lower cytotoxic potency; therefore, a concentration corresponding approximately to its IC_25_ value was selected to allow the evaluation of its potential chemosensitizing activity under subcytotoxic conditions.

The chemosensitizing potential of *E. heterophyllum* extracts in A549 cells was evaluated using the crystal violet assay (Feoktistova et al., 2016). A549 cells (5 × 10^3^ cells/well) were seeded in 96-well plates and treated for 48 h with increasing concentrations of PTX in the absence or presence of fixed concentrations of each extract. The extracts were added simultaneously to the same wells at subcytotoxic concentrations corresponding to the IC_10_ values of the hexane and acetone extracts (10 μg/mL) and the IC_25_ value of the hydroalcoholic extract (50 μg/mL). PTX alone and extract-alone groups were included as controls. Cell viability was determined by crystal violet staining (Sigma-Aldrich), and absorbance was measured at 570 nm using an ELISA microplate reader. Concentration–response curves and IC_50_ values were obtained by nonlinear regression analysis using GraphPad Prism software. Experiments were performed in triplicate in three independent assays.

### 2.8 Immunofluorescence

The expression of P-gp was analyzed in A549 lung cancer cells, for which 2 × 10^4^ A549 cells were seeded in 24-well culture plates containing round coverslips. The cells were treated with the previously determined IC_50_ for each extract for 24 h at 37 °C and 5% CO_2_.

Following treatment, cells were fixed with 4% paraformaldehyde (PFA) in PEM buffer (100 mM PIPES pH 6.9, 5 mM EGTA, 2 mM MgCl_2_) for 15 min. Subsequently, a solution of PFA/0.1 M NaHCO_3_ was added and incubated for 45 min at room temperature; the addition of NaHCO_3_ served to stabilize the pH during extended fixation and optimize the preservation of P-gp antigenic epitopes prior to permeabilization. After each reagent addition, the media was aspirated, and the cells were thoroughly washed with 1X PBS. Cells were then treated with 0.1% Triton X-100 (Sigma Aldrich) in 1X PBS for 10 min for permeabilization. To prevent and monitor non-specific binding, a negative control omitting the primary antibody was used, which showed no detectable fluorescence. Cells were incubated with the monoclonal mouse anti-P-gp primary antibody (clone sc-55510, Santa Cruz Biotechnology, Dallas, TX, USA; validated by the manufacturer for immunofluorescence) at a 1:100 dilution overnight at 4 °C. After washing, the secondary antibody (anti-mouse Alexa 647 1:1000, A-21235, Molecular Probes, Thermo Fisher Scientific, Inc., Waltham, MA, USA) was added and incubated for 1 hour at 37 °C to favor binding kinetics in this specific cell model.

The cells were then stained with DAPI (Sigma-Aldrich, St. Louis, MO, USA, D9542), for 10 min. Finally, the coverslips were mounted using SignalStain® Aqueous Mounting Medium (#27290, Cell Signaling Technology) to prevent photobleaching. The immunofluorescence staining procedure was adapted from previously reported protocols (Alejandre-García et al., 2015; Antúnez-Mojica et al., 2019).

Samples were visualized on an Olympus FluoView FV1000 confocal microscope system coupled to an inverted IX81 Olympus microscope with a UPlanSApo 40× (numeric aperture 0.75) objective.

For the semi-quantitative analysis, fluorescence intensity was quantified using ImageJ software to determine Units Relative Fluorescence (URF). The experiments were performed in triplicate in three independent experiments.

### 2.9 Cellular doxorubicin retention assay

To evaluate the functional modulation of the P-glycoprotein (P-gp) efflux system, a doxorubicin retention assay was performed using doxorubicin as a fluorescent P-gp substrate and verapamil as a positive control, following previously described approaches with minor modifications (Sánchez-Carranza et al., 2019). Since A549 cells exhibit functional basal expression of P-gp, they constitute a suitable model for evaluating the inhibitory potential of the extracts (Peng et al., 2021; Salomon and Ehrhardt, 2011).

Cells (2.5 × 10^5^) were cultured for 24 h and subsequently pre-incubated for 2 h at 37 °C with DMEM medium, 20 µM verapamil (positive control), or 100 μg/mL of the hexane, acetone, or hydroalcoholic extracts. A concentration of 100 μg/mL was selected for all extracts to ensure maximum inhibitory effect on the efflux pump during this short-term functional assay. Subsequently, the cells were treated with 20 µM doxorubicin (Sigma-Aldrich), which acts as an intrinsic fluorescent substrate for P-gp, for 4 h at 37 °C. After treatment, the culture medium was removed, and the cells were trypsinized and harvested, followed by a double wash with cold PBS to completely eliminate any remaining extracellular doxorubicin. Intracellular fluorescence intensity, which correlates with doxorubicin retention, was analyzed by flow cytometry using a BD FACSLyric™ flow cytometer (Becton Dickinson, San Jose, CA, USA)

For each sample, 10,000 events were acquired using an excitation wavelength of 488 nm and an emission channel FL2 (∼575 nm). The gating strategy consisted of selecting the main cell population based on size (FSC) and complexity (SSC) properties, excluding cell debris. All experiments were performed in triplicate in three independent trials.

### 2.10 Total phenolic content

The TPC (total phenolic content) quantification was carried out by the Folin-Ciocalteu method (Teixeira et al., 2016). Briefly, an aliquot of 28 μL of acetone extract dissolved in 75:25 H_2_O:MeOH and 100% water of hydroalcoholic extract (0.9 mg/mL) were mixed with 42 μL of Folin-Ciocalteu reagent (1N); the mixture was keeping on orbital shaker at 80 ± 1 rpm and incubated at room temperature in the dark for 5 min. Then, 42 μL of a Na_2_CO_3_ solution (20%; w/v) was added and 168 μL of distilled water, incubated for 30 min under the same conditions. Samples were immediately read against a blank on a Multiscan Go spectrophotometer, at a wavelength of 760 nm. The TPC in the samples was calculated by comparing the optical density (OD) of a gallic acid standard curve (5–100 μg/mL; R2 = 0.9998; y = 0.0097x + 0.0656). The results are expressed as mg of gallic acid equivalents per g of dry extract mg). GAE/g extract. All determinations were made in triplicate (n = 3) and are reported as the mean ± standard deviation.

### 2.11 Determination of flavonoid content

Flavonoid content was determined using aluminum chloride assay according to (Seifu et al., 2017). Briefly, an aliquot (28 μL) of the extract was mixed with 112 μL of distilled water, also 8.4 μL of 5% NaNO_2_ was added. After 5 min of incubation, 8.4 μL of 10% AlCl_3_ was added. After 1 min, 56 μL of 1M NaOH was added and the volume was a adjusted to 280 μL with 67.2 μL of distilled water. After 10 min, the absorbance of the resulting solution was measured at 510 nm. Catechin was used as a standard curve (5–100 μg/mL; *R* ^2^ = 1; y = 0.0024x + 0.046) to express total flavonoids contents of samples as mg catechin equivalent per g of sample (mgECA/g sample). All the samples were analyzed in triplicate.

### 2.12 Gas chromatography/mass spectrometry

The extracts of *E. heterophyllum* were analyzed at the Mass Spectrometry Laboratory of the Institute of Chemistry at the National Autonomous University of México. The analysis was performed by gas chromatography-mass spectrometry (GC-MS) using an Agilent Technologies 7890A gas chromatograph (Agilent Technologies, Santa Clara, CA, USA) coupled to a Jeol GC Mate II mass spectrometer. Separation was performed on a 30 m long NP-5 capillary column with an internal diameter of 0.32 mm and a film thickness of 0.25 µm (5% diphenyl/95% dimethylpolysiloxane). Helium was used as the carrier gas at a constant flow rate of 1 mL/min. The injector temperature was maintained at 300 °C and 1 µL of sample was injected in split mode (50:1). Samples were dissolved in hexane at a concentration of 1 mg/mL prior to injection. The oven temperature programmed started at 40 °C (held for 1 min) and increased to 300 °C at a rate of 8 °C/min. For detection, the mass spectrometer operated in electron impact ionization mode at 70 eV, recording a mass range of m/z 10 to 600 with a scan time of 1.3 s. The determination of the metabolic composition was performed by identity comparison, selecting those metabolites that met a match factor ≥80% according to the NIST (National Institute of Standards and Technology) database.

### 2.13 Statistical analysis

Statistical analyses were performed using GraphPad Prism 8.0 software (GraphPad Software Inc., La Jolla, CA, USA). Concentration–response curves and IC_50_ values were obtained by nonlinear regression analysis. Data are presented as mean ± SD of three independent experiments. Statistical significance among groups was determined using one-way ANOVA followed by Dunnett’s multiple comparisons test against the PTX treated group. Differences were considered statistically significant at *p ≤ 0.05, **p ≤ 0.01, ***p ≤ 0.001 and ****p ≤ 0.0001.

## 3 Results

### 3.1 Extraction yield of *Eryngium heterophyllum* extracts

To establish the phytochemical basis underlying the biological effects of *E. heterophyllum* extracts, extraction yield and phytochemical profiling were first evaluated.

The extraction yields obtained from *E. heterophyllum* leaves and stems varied according to solvent polarity. The yield percentage for the hexane extract was 1% (40 g), for the acetone extract 2% (80 g) and 5.5% (330 g) for the hydroalcoholic extract.

The hydroalcoholic extract showed the highest yield, followed by the acetone extract and the hexane extract. These differences reflect the heterogeneous nature of the plant matrix and the preferential extraction of polar and semi-polar metabolites by hydroalcoholic and acetone solvents, respectively.

### 3.2 Cytotoxic activity and IC_50_ values of *Eryngium heterophyllum* extracts in A549 cells

**FIGURE 1:**
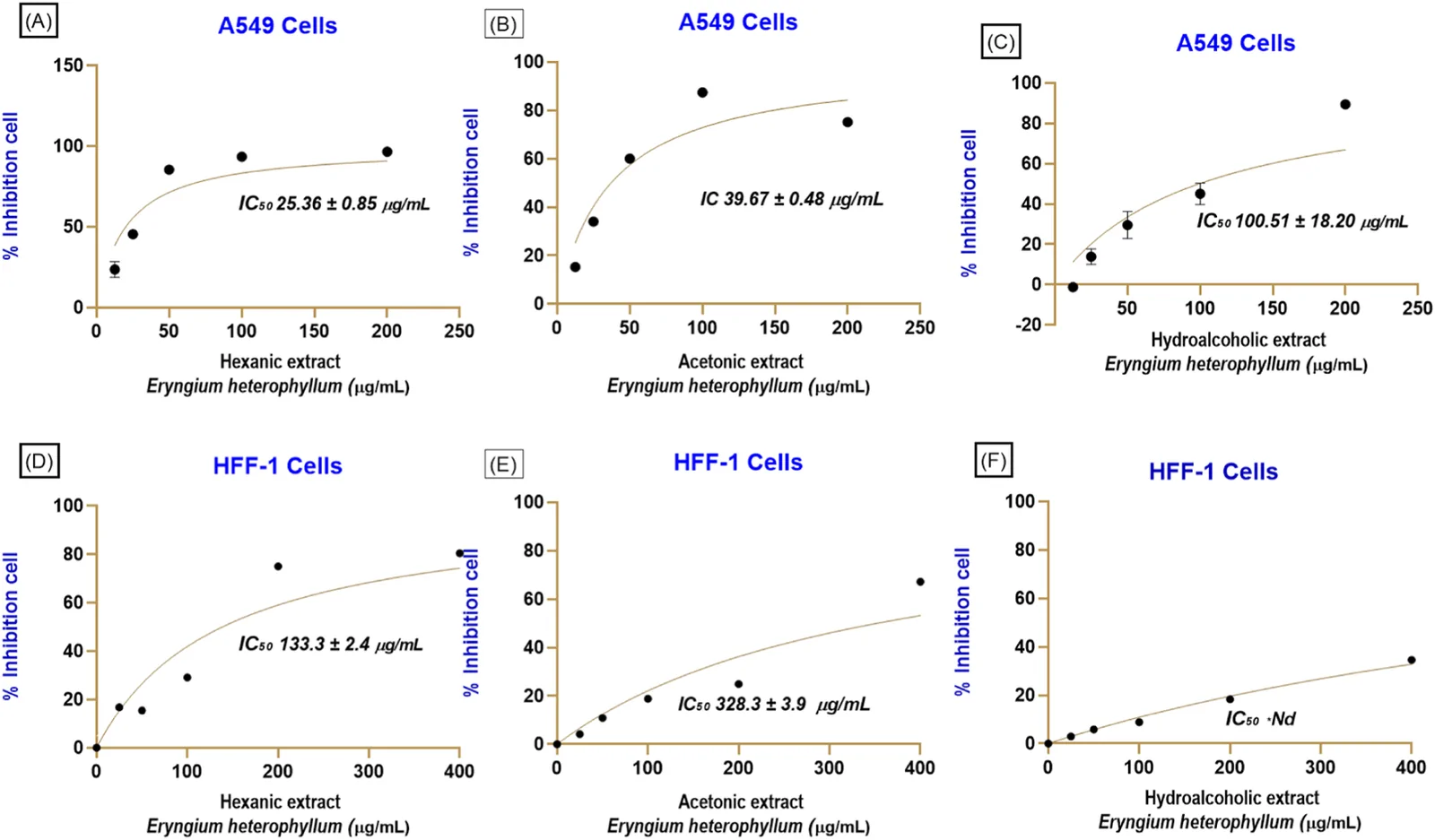
Concentration–response curves of *Eryngium heterophyllum* extracts in A549 lung cancer cells and HFF-1 human foreskin fibroblasts. A549 cells **(A–C)** and HFF-1 cells **(D–F)** were exposed for 48 h to increasing concentrations of the hexane **(A,D)** acetone **(B,E)** and hydroalcoholic **(C,F)** extracts. Cell viability was determined using the crystal violet assay and expressed as percentage of growth inhibition relative to untreated controls. IC_50_ values were calculated by nonlinear regression analysis using GraphPad Prism software. Data represent the mean ± SD of three independent experiments performed in triplicate.

The cytotoxic effects of *E. heterophyllum* extracts on A549 lung cancer cells and HFF-1 human foreskin fibroblasts were evaluated using concentration-response assays. The cytotoxic potency of each extract was determined by calculating the median inhibitory concentration (IC_50_) values ​​in both cell lines (Figures 1). The hexane extract showed the highest cytotoxic activity against A549 cells (IC_50_ = 25.1 ± 1.1 μg/mL), followed by the acetone extract (IC_50_ = 39.7 ± 1.3 μg/mL), while the hydroalcoholic extract showed the lowest activity (IC_50_ = 100.5 ± 6.1 μg/mL). It is worth noting that the extracts were less toxic to HFF-1 fibroblasts, resulting in favorable selectivity indices and suggesting preferential activity against cancer cells.

**FIGURE 2:**
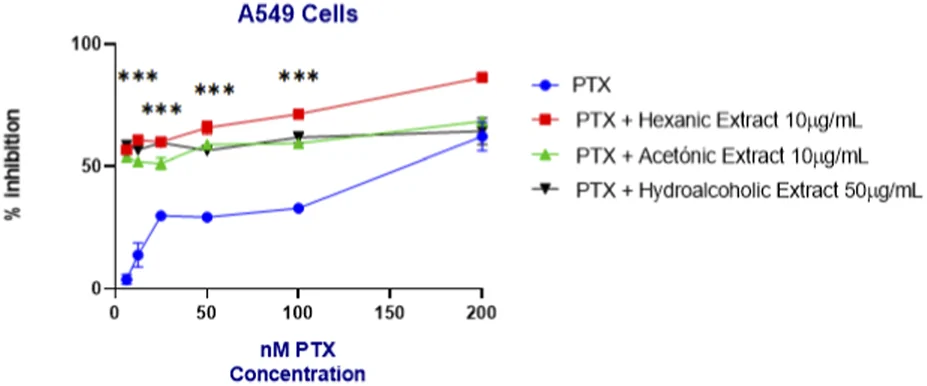
Effect of *Eryngium heterophyllum* extracts on PTX -mediated growth inhibition in A549 cells. Cells were treated with increasing concentrations of PTX alone or in combination with hexane extract (10 μg/mL), acetone extract (10 μg/mL), or hydroalcoholic extract (50 μg/mL) for 48 h. Growth inhibition was expressed as percentage relative to untreated control cells. Data are expressed as mean ± SD of three independent experiments. Statistical analysis was performed using one-way ANOVA followed by Dunnett’s multiple comparisons test against the PTX-treated group. Asterisks (***) indicate statistically significant differences (*p* ≤ 0.001) between combination treatments and PTX alone at the corresponding concentration.

The selectivity index (SI), defined as the ratio between IC_50_ values in non-cancer and cancer cells, was calculated for each extract (Table 1). An SI ≥ 2 was considered indicative of selective cytotoxicity toward cancer cells.

**TABLE 1:** Cytotoxic activity and selectivity of *Eryngium heterophyllum* extracts and PTX in A549 lung cancer cells and human foreskin fibroblasts (HFF-1). IC_50_ values are expressed as mean ± standard deviation (n = 3).

Sample	Cells		SI
	A549	HFF-1	
	(µg/mL)		
Hexane extract	25.1 ± 1.1	133.3 ± 2.4	5.3
Acetone extract	39.7 ± 1.3	328.3 ± 3.9	8.2
Hydroalcoholic extract	100.5 ± 6.1	>400	>3.9
PTX; nM	166.48 ± 12.78	993.5 ± 5.1	5.9

A549, Lung cancer; HFF-1, immortalized fibroblasts. For the hydroalcoholic extract, the SI is reported as ≥3.9 because the IC_50_ in HFF-1 cells was not reached within the tested concentration range.

In terms of selectivity, the acetone extract displayed the highest selectivity index (SI = 8.2), followed by PTX (SI = 6.8) and the hexane extract (SI = 5.3), indicating preferential cytotoxicity toward cancer cells compared with normal fibroblasts. Notably, although the hydroalcoholic extract was less potent, no significant cytotoxicity was observed in HFF-1 cells at the highest concentration tested (IC_50_ > 400 μg/mL), resulting in an estimated selectivity index greater than 3.9.

Collectively, these results demonstrate that *Eryngium heterophyllum* extracts exert intrinsic antiproliferative activity in A549 cells and support their evaluation as potential chemosensitizing agents.

### 3.3 *Eryngium heterophyllum* extracts modify sensitivity to PTX

To evaluate the potential chemosensitizing effect of *E. heterophyllum* extracts on PTX activity, combination experiments were performed in A549 lung cancer cells. Prior to these assays, extract concentrations with minimal intrinsic cytotoxicity were selected to allow a clearer assessment of their modulatory effect on PTX response.

Based on the concentration–response curves shown in Figure 1, subcytotoxic concentrations (IC_10_–IC_25_ range) were selected for subsequent combination assays with PTX to minimize the intrinsic cytotoxic effect of the extracts. The chosen concentrations were 10 μg/mL for the hexane and acetone extracts and 50 μg/mL for the hydroalcoholic extract. Although the extracts exhibited intrinsic antiproliferative activity, these concentrations were selected because they produced only modest effects on cell viability (approximately 10% for the hexane extract, 10% for the acetone extract, and 25% for the hydroalcoholic extract) when administered alone, thereby allowing the assessment of their potential chemosensitizing effect in combination with PTX.

As shown in Figure 2, PTX alone induced a concentration-dependent increase in the inhibition of A549 cell proliferation. Notably, co-treatment with *E. heterophyllum* extracts significantly enhanced the antiproliferative effect of PTX at all tested concentrations. Among the combinations, the hexane extract produced the greatest potentiation of PTX activity, followed by the acetone and hydroalcoholic extracts. Furthermore, the presence of the extracts shifted the concentration–response curve upward compared with PTX alone, indicating a greater inhibitory effect on proliferation and suggesting an enhanced cytotoxic response in A549 cells.

To quantitatively confirm the potentiation observed in the combination assays, the IC_50_ of PTX was determined in the presence and absence of *Eryngium heterophyllum* extracts (Table 2). PTX alone exhibited an IC_50_ of 166.48 ± 12.78 nM in A549 lung cancer cells. Co-treatment with the extracts markedly enhanced PTX potency, as evidenced by a pronounced reduction in IC_50_ values. The hexane extract produced the strongest effect, decreasing the IC_50_ to 12.261 ± 0.336 nM. Similarly, the hydroalcoholic and acetone extracts reduced the IC_50_ to 13.057 ± 0.495 nM and 15.682 ± 0.139 nM, respectively. Overall, the combination treatments reduced the effective concentration of PTX by approximately 10–14 fold compared with PTX alone, confirming the potent chemosensitizing capacity of *E. heterophyllum* extracts in A549 lung cancer cells.

**TABLE 2:** IC_50_ values of paclitaxel alone and in combination with *Eryngium heterophyllum* extracts in A549 cells.

Treatments	IC_50_ values (nM)
PTX	166.48 ± 12.78
PTX + Hexane extract (10 µg/mL)	12.26 ± 0.33
PTX + Acetone extract (10 µg/mL)	15.68 ± 0.13
PTX + Hydroalcoholic extract (50 µg/mL)	13.05 ± 0.49

These findings demonstrate a pronounced chemosensitizing effect of the extracts, with the hexane extract producing the greatest enhancement of PTX cytotoxicity in A549 cells.

The combination index (CI), estimated according to the Chou–Talalay method, revealed a clear synergistic interaction between paclitaxel and the extracts of *Eryngium heterophyllum* in A549 cells.

The CI was calculated using the classical equation:

$$\text{CI}=\frac{D1}{{\text{Dx}}_{1}}+\frac{D2}{{\text{Dx}}_{2}}$$

Where:

- D_1_ represents the concentration of PTX in the combination (IC_50_ of the combination).
- Dx_1_ represents the IC_50_ of PTX alone.
- D_2_ represents the concentration of the extract used in the combination.
- Dx_2_ represents the IC_50_ of the extract alone.

According to standard criteria, CI < 1 indicates synergism, CI = 1 an additive effect, and CI > 1 antagonism. As shown in Table 3, all evaluated combinations produced CI values below unity, confirming a synergistic interaction.

**TABLE 3:** Combination Index (CI) and synergistic interactions between PTX and *Eryngium heterophyllum* extracts.

Treatment combination	D1 (PTX, nM)	Dx1 (PTX IC_50_, nM)	D2 (Extract, µg/mL)	Dx2 (Extract IC_50_, µg/mL)	CI value	Interaction
PTX + Hexane extract	12.261	166.48	10	25.1	0.47	Synergism
PTX + Acetone extract	15.682	166.48	10	39.7	0.35	Strong synergism
PTX + Hydroalcoholic extract	13.057	166.48	50	100.5	0.58	Moderate synergism

Notably, the acetone extract exhibited the strongest synergistic effect (CI = 0.35), followed by the hexane extract (CI = 0.47) and the hydroalcoholic extract (CI = 0.58). Importantly, the extracts were tested at sub-cytotoxic concentrations, indicating that the enhanced antiproliferative effect primarily reflects true chemosensitization rather than additive toxicity. Collectively, these findings demonstrate that *E. heterophyllum* extracts markedly potentiate PTX efficacy in A549 cells and support their potential role as adjuvant chemosensitizing agents.

### 3.4 Morphological changes induced by *Eryngium heterophyllum* Extract–PTX combinations in A549 cells

**FIGURE 3:**
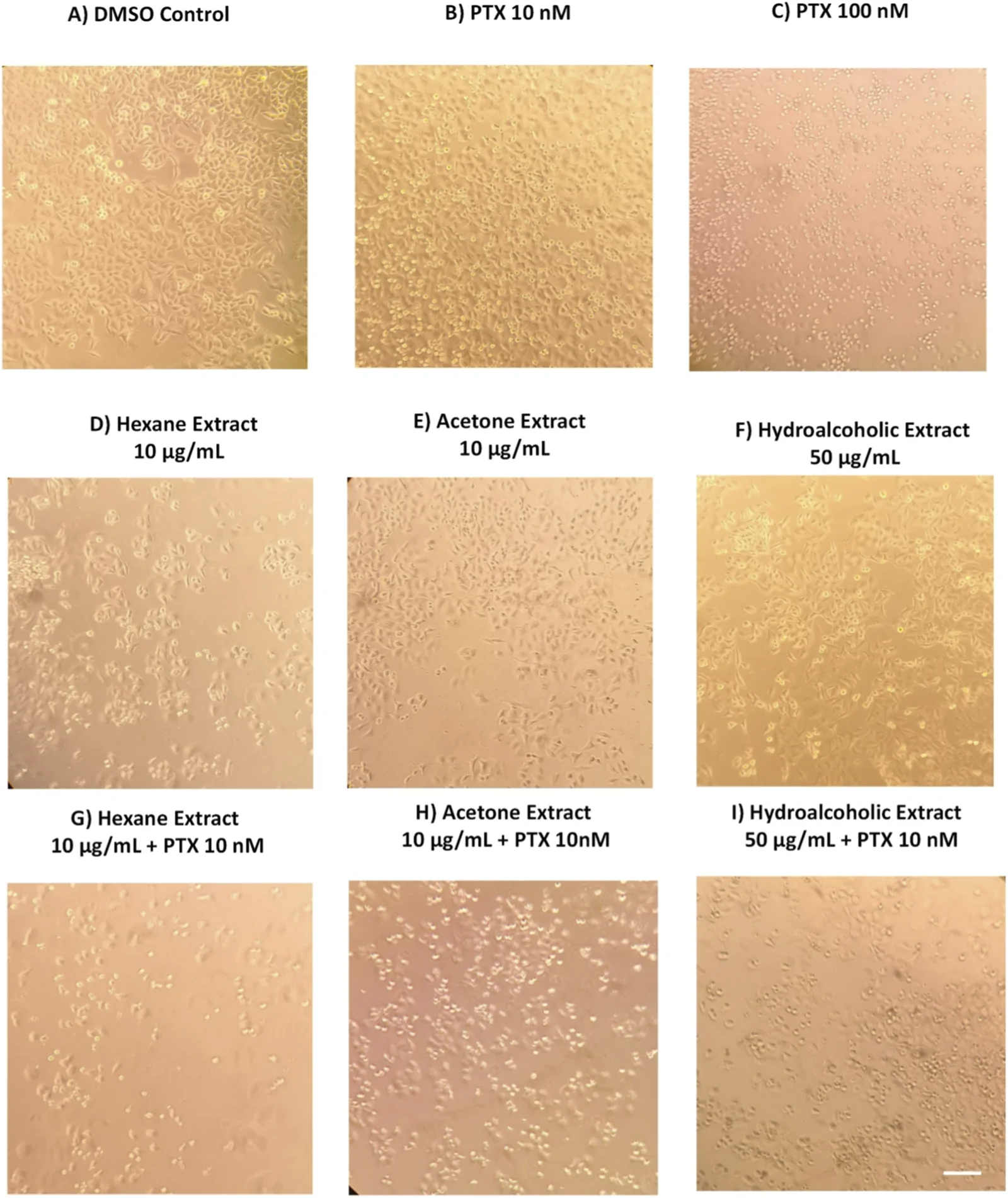
A549 cells evaluated by bright-field optical microscopy under different experimental conditions. Images were acquired at a magnification of ×10 after 24 h of treatment. **(A)** Negative control (vehicle); **(B,C)** Positive control (PTX, 10 nM and 100 nM); **(D–F)** Cells treated with individual extracts (hexane extract, 10 μg/mL; acetone extract, 10 μg/mL; hydroalcoholic extract, 50 μg/mL); **(G–I)** Cells treated with combined treatments (hexane extract + PTX; acetone extract + PTX; hydroalcoholic extract + PTX). Representative images from three independent experiments are shown. All images are shown at the same scale (scale bar = 100 µm).

The micrographs on Figure 3 illustrate the morphological changes in A549 cells after treatment with PTX, *E. heterophyllum* extracts, and their respective combinations. In the control condition (top left), the cells showed characteristic epithelial morphology, with high confluence, an elongated-polygonal shape, and firm adherence to the culture substrate, indicating a normal proliferative state.

Treatment with the extracts alone (hexane, acetone, and hydroalcoholic), as well as with low-concentration PTX, resulted in a largely conserved cell monolayer. While occasional rounded cells and slight reductions in cell density were observed, most cells remained adherent and maintained a morphology comparable to that of the control group, consistent with the use of subcytotoxic concentrations. In contrast, the combined treatments induced pronounced morphological alterations. A marked increase in rounded cells, loss of the typical spindle-shaped morphology, decreased cell adhesion, and the presence of floating cells and cellular debris were observed. These characteristics are typical of cell death and were comparable to those observed in cells treated with 100 nM PTX.

Taken together, the morphological evidence supports the notion that the combination of *Eryngium heterophyllum* extracts with PTX potentiates cell damage compared to the individual treatments, which is consistent with the potentiating (chemosensitizing) effect previously observed in viability assays.

Morphological changes in A549 cells after treatment with *E. heterophyllum* extracts and PTX. Images are representative of three independent experiments.

### 3.5 Nuclear fragmentation induced by extract–PTX combinations in A549 cells

**FIGURE 4:**
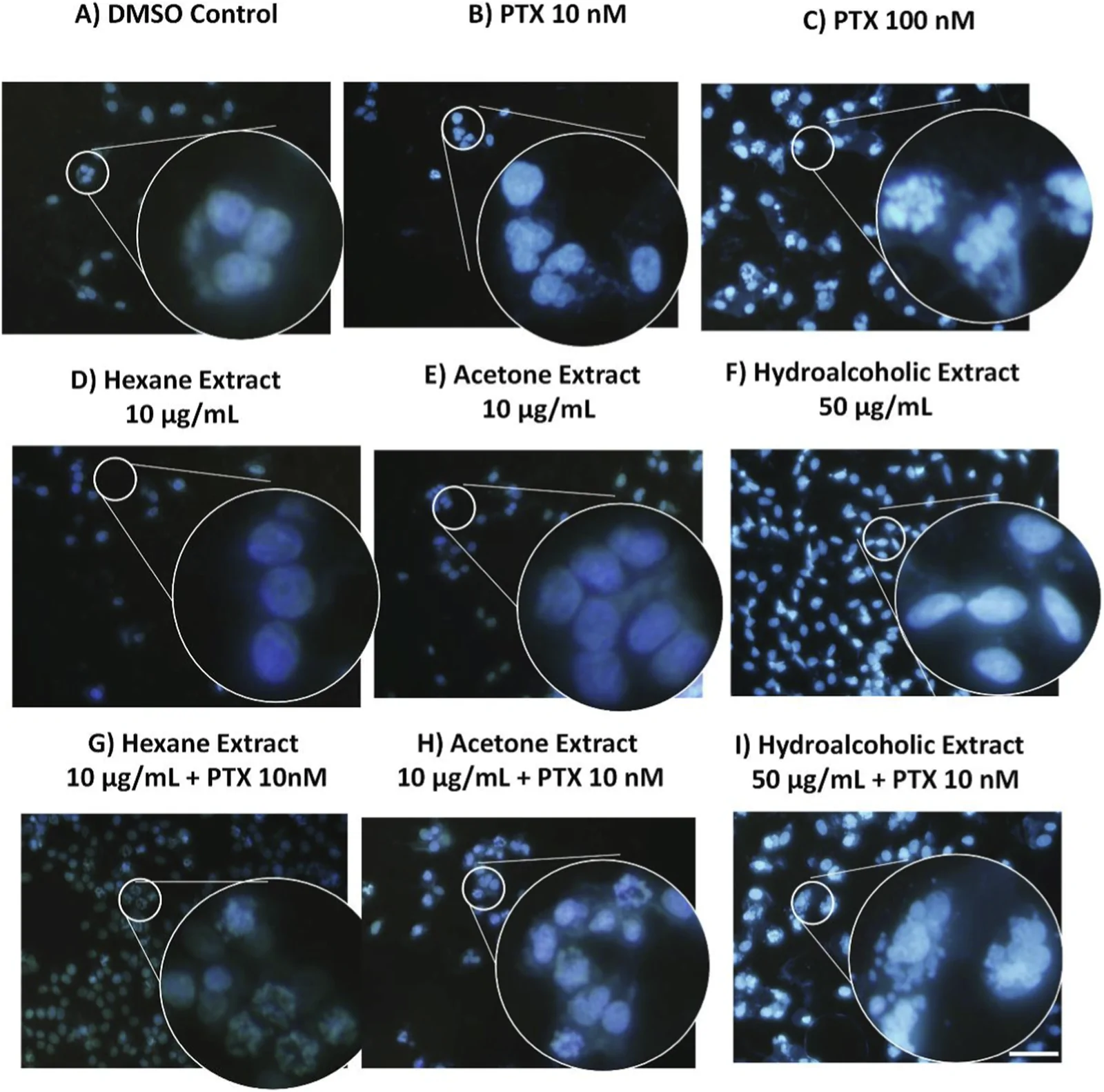
Representative fluorescence micrographs of A549 lung cancer cells stained with DAPI after 24 h of treatment. **(A)** Negative control (vehicle); **(B,C)** Positive control (PTX, 10 and 100 nM, respectively); **(D–F)** Cells treated with *Eryngium heterophyllum* extracts: **(D)** hexane extract (10 μg/mL); **(E)** acetone extract (10 μg/mL); **(F)** hydroalcoholic extract (50 μg/mL). **(G–I)** Cells treated with combinations of each extract with paclitaxel (10 nM). Representative images from three independent experiments are shown. All images are shown at the same scale (scale bar = 50 µm).

Given the pronounced enhancement of PTX cytotoxicity observed in the combination assays, nuclear fragmentation was subsequently evaluated by DAPI staining as a morphological indicator commonly associated with apoptosis. A549 cells were treated with *E. heterophyllum* extracts, PTX, and their respective combinations (Figure 4).

Treatment with hexane (10 μg/mL), acetone (10 μg/mL), or hydroalcoholic (50 μg/mL) extracts alone did not induce evident nuclear alterations compared with control cells. Similarly, PTX at 10 nM produced slight nuclear alterations compared with control cells. In contrast, cells treated with the combinations of extracts and PTX exhibited more evident chromatin condensation and nuclear fragmentation. These morphological features were consistently observed across all combination treatments, with a higher frequency of condensed and fragmented nuclei compared with the individual treatments, exhibiting a nuclear alteration pattern comparable to that observed with PTX at 100 nM. Since paclitaxel is known to induce apoptosis-associated morphological changes, these findings may suggest a possible potentiation of PTX-associated effects by the extracts. Future studies including Annexin V/PI staining, caspase activation, or TUNEL assays would help to further characterize the possible involvement of apoptotic cell death mechanisms.

### 3.6 Immunofluorescence for P-gp

To further investigate the mechanism underlying the enhanced cytotoxic response observed in the combination treatments, the effect of *Eryngium heterophyllum* extracts on P-gp expression was evaluated using confocal microscopy. Given the central role of P-gp in MDR, this analysis aimed to determine whether modulation of this efflux transporter could contribute to the increased sensitivity of A549 cells to PTX.

**FIGURE 5:**
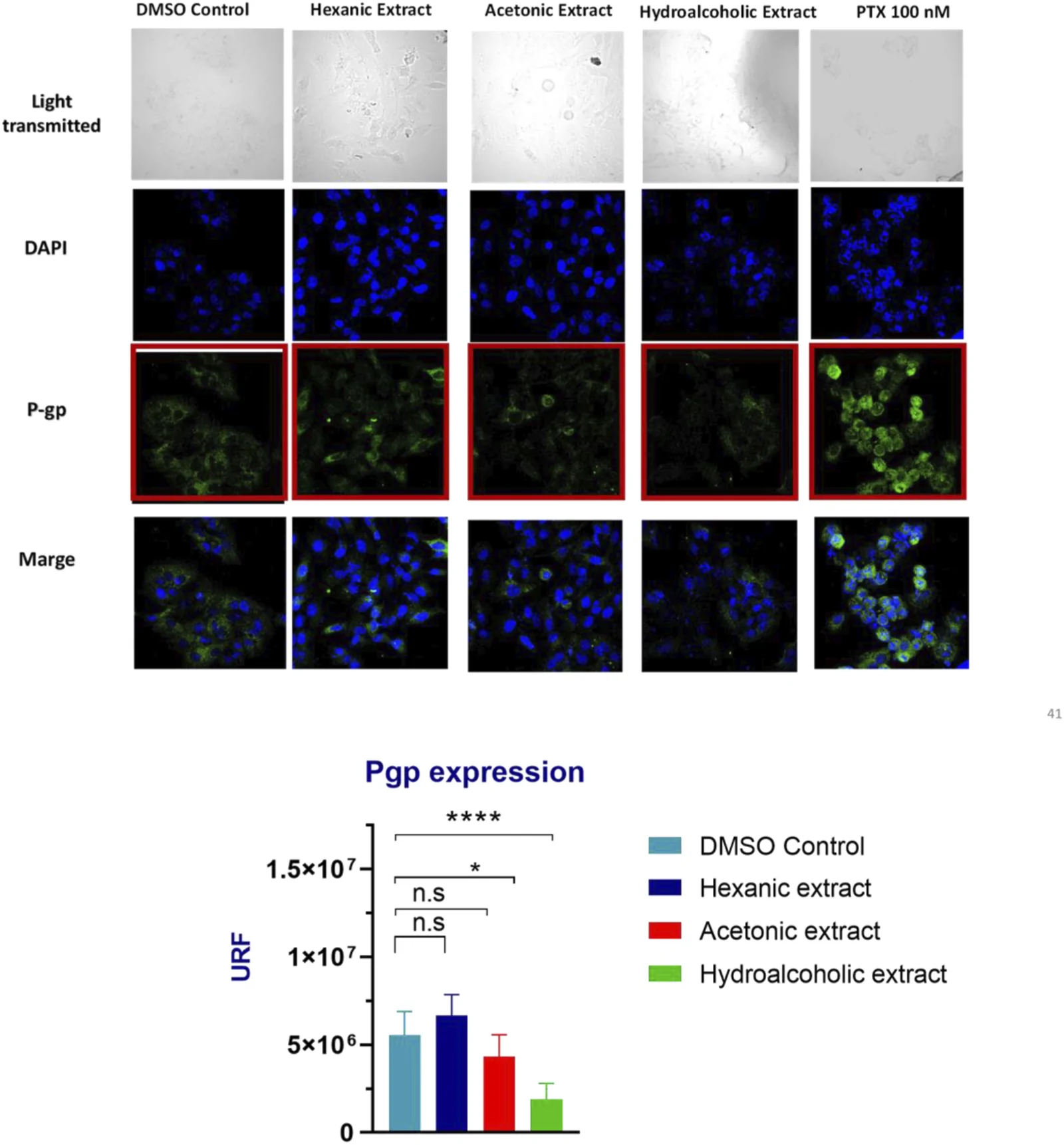
Confocal laser scanning microscopy analysis of P-gp expression in A549 lung cancer cells treated with *Eryngium heterophyllum* extracts and PTX. (Top) Representative bright-field images, DAPI-stained nuclei (blue), P-gp immunofluorescence (green), and merged images are shown. (Bottom) Quantitative analysis of P-gp expression expressed as Units Relative Fluorescence (URF). Data are presented as mean ± SD of three independent experiments. Statistical significance is indicated as follows: ns, not significant (p ≥ 0.05); *p < 0.05; **p < 0.01; ***p < 0.001; ****p < 0.0001.

Confocal laser scanning microscopy revealed treatment-dependent differences in P-gp expression and cell morphology (Figure 5). Bright-field images showed mild alterations in cell density and adhesion between the treated groups. DAPI (blue) staining allowed clear visualization of cell nuclei, which appeared predominantly intact in control and extract-treated cells, while PTX treated cells exhibited nuclear alterations consistent with PTX induced cell damage.

Regarding P-gp immunofluorescence (green), control cells showed a baseline fluorescence signal. Treatment with PTX produced the highest fluorescence intensity, suggesting an adaptive increase in P-gp expression following drug exposure. In contrast, treatment with the extracts resulted in lower fluorescence levels, with the most pronounced reduction observed with the hydroalcoholic extract. The hexane and acetone extracts also showed intermediate fluorescence intensities compared to the PTX treated group.

These qualitative observations were consistent with the fluorescence quantification analysis, which demonstrated reduced P-gp expression in cells treated with the extracts compared to cells treated with PTX. Taken together, these findings suggest that *E. heterophyllum* extracts, particularly the hydroalcoholic fraction, could modulate P-gp expression in A549 cells.

### 3.7 Doxorubicin retention assay

The functional activity of P-gp was further assessed using a doxorubicin retention assay in A549 cells. Although A549 cells are generally considered drug-sensitive, several studies have demonstrated that they exhibit basal expression of functional P-gp, making them a suitable model for evaluating the initial modulation of drug efflux mechanisms by potential chemosensitizing agents (Peng et al., 2021; Salomon and Ehrhardt, 2011).

**FIGURE 6:**
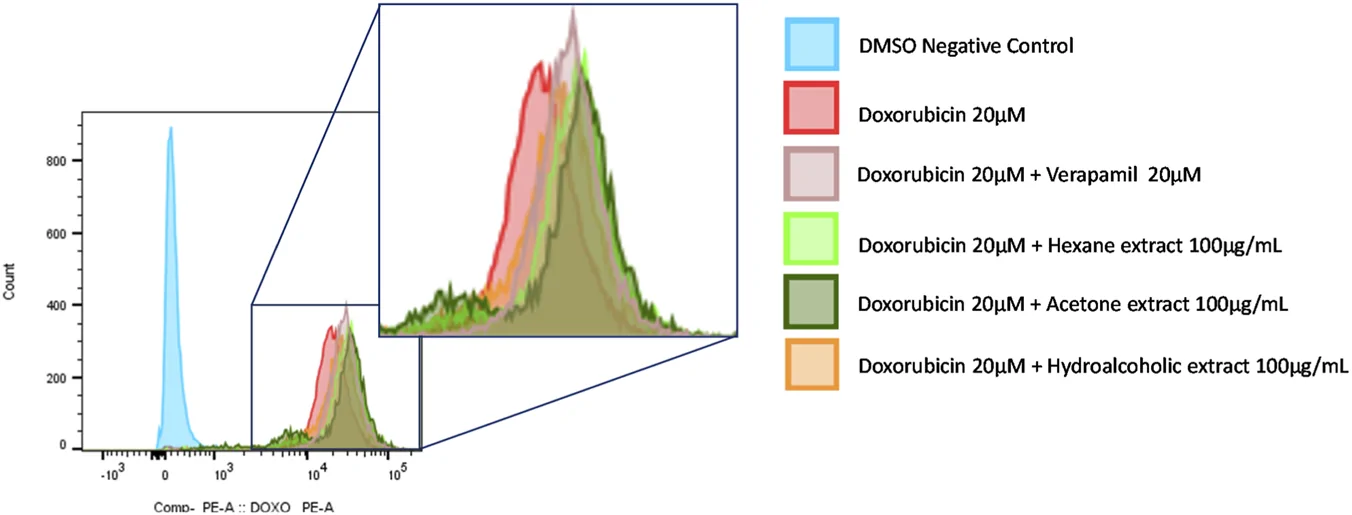
Effect of *Eryngium heterophyllum* extracts on doxorubicin retention in A549 cells. Intracellular accumulation of doxorubicin was evaluated by flow cytometry after treatment with doxorubicin alone, verapamil (positive control), or the hexane, acetone, and hydroalcoholic extracts. Representative images from three independent experiments are shown.

Control cells exposed to doxorubicin alone showed a fluorescence distribution. As expected, treatment with the positive control verapamil resulted in a clear rightward shift in fluorescence intensity, indicative of increased intracellular accumulation of doxorubicin due to inhibition of P-gp-mediated transport. Importantly, cells treated with hexane, acetone, and hydroalcoholic extracts also showed a marked rightward shift in fluorescence peaks compared to doxorubicin alone, reflecting increased intracellular retention of the drug (Figure 6). The magnitude and pattern of the shift were comparable to those observed with verapamil, suggesting that all three extracts interfere with P-gp efflux activity in A549 cells.

### 3.8 Total polyphenol and flavonoid content of the acetone and hydroalcoholic extract

**FIGURE 7:**
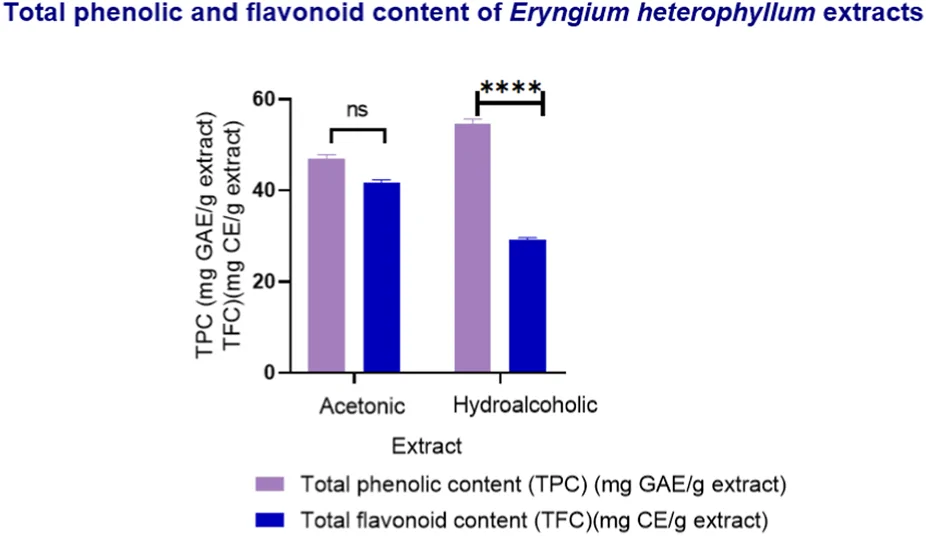
Total phenolic content (TPC) and total flavonoid content (TFC) of *Eryngium heterophyllum* extracts. TPC was expressed as milligrams of gallic acid equivalents per Gram of dry extract (mg GAE/g extract), whereas TFC was expressed as milligrams of catechin equivalents per Gram of dry extract (mg CE/g extract). The hydroalcoholic extract exhibited the highest phenolic. Data are presented as mean ± SD of three independent determinations (n = 3). Statistical analysis was performed using an unpaired Student’s t-test. Statistical significance between the acetone and hydroalcoholic extracts is indicated as follows: ns, not significant (*p* ≥ 0.05); *p < 0.05; **p < 0.01; ***p < 0.001; ****p < 0.0001. content, while the acetone extract showed the highest flavonoid content. Values represent the mean ± SD of three independent determinations (n = 3).

The total phenolic content (TPC) and total flavonoid content (TFC) of the acetone and hydroalcoholic extracts are presented in Table 4 and Figure 7. The hydroalcoholic extract exhibited the highest TPC, whereas the acetone extract showed the highest TFC. These results indicate that solvent polarity influenced the extraction efficiency of different classes of phytochemicals, leading to distinct phenolic and flavonoid profiles in each fraction.

**TABLE 4:** Total phenolic and flavonoid content of *Eryngium heterophyllum* extracts. Data are presented as mean ± standard deviation (n = 3). Calibration curves and coefficients of determination (*R* ^2^) are shown for each assay.

Parameter	Acetone extract	Hydroalcoholic extract
Total phenolic content (TPC) (mg GAE/g extract)	46.97 ± 0.82	54.68 ± 0.94
Calibration curve (TPC)	y = 0.0097x + 0.0656	y = 0.0097x + 0.0656
*R* ^2^ (TPC)	0.99	0.99
Total flavonoid content (TFC) (mg CE/g extract)	41.65 ± 0.62	29.13 ± 0.49
Calibration curve (TFC)	y = 0.0024x + 0.046	y = 0.0024x + 0.046
*R* ^2^ (TFC)	0.99	0.99

Phytochemical profiling revealed that both acetone and hydroalcoholic extracts were enriched in phenolic metabolites compared with the hexane extract. In contrast, flavonoids were more abundant in the acetone fraction, suggesting a preferential extraction of moderately polar flavonoid metabolites by acetone. This observation is particularly relevant because phenolic metabolites and flavonoids have been widely associated with antioxidant, antiproliferative, and chemosensitizing activities. Therefore, the differential distribution of these metabolites among the extracts may contribute, at least in part, to the biological activities observed in the present study.

### 3.9 Gas chromatography/mass spectrometry analysis

**FIGURE 8:**
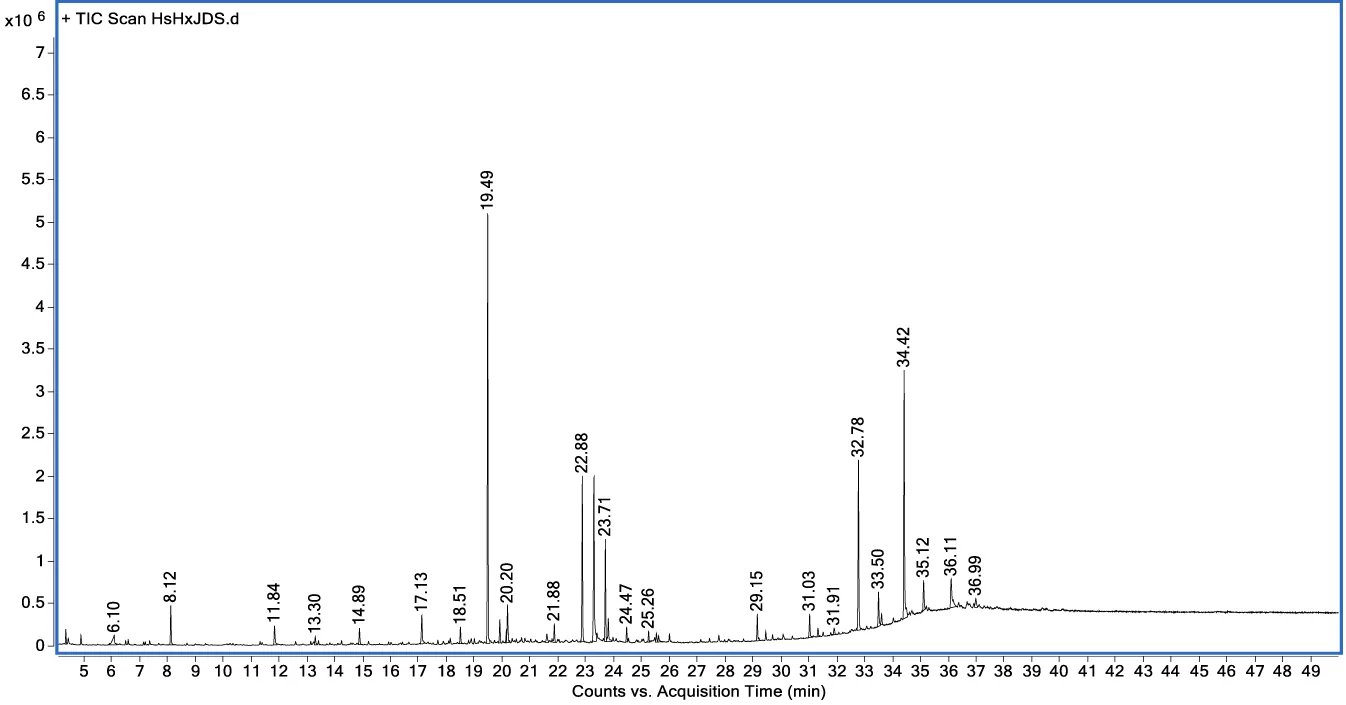
GC-MS chromatogram of the hexane extract from *Eryngium heterophyllum*.

**FIGURE 9:**
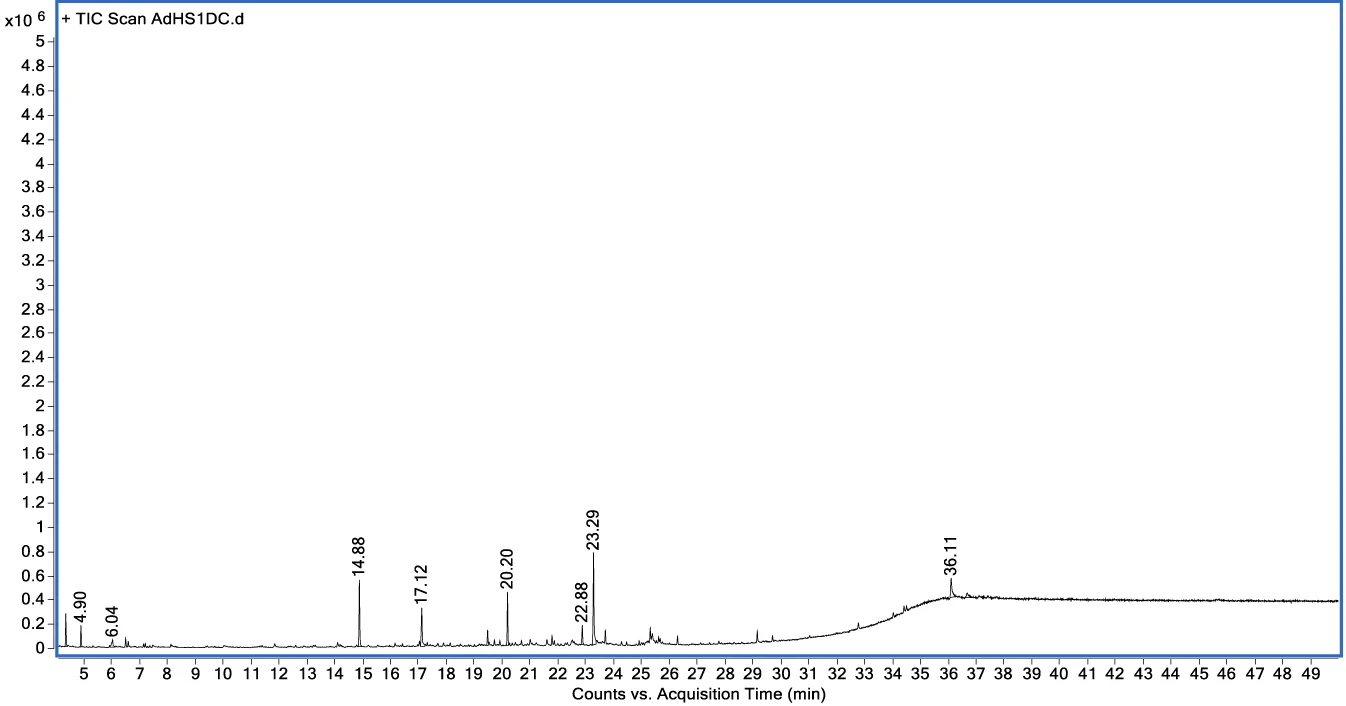
GC-MS chromatogram of the acetone extract from *Eryngium heterophyllum*.

Preliminary chemical characterization of *Eryngium heterophyllum* extracts was performed by gas chromatography–mass spectrometry (GC–MS) analysis to identify metabolites potentially associated with the observed cytotoxic and paclitaxel-sensitizing activities. Compound identification was achieved through analysis of mass fragmentation patterns and comparison with the NIST spectral library. Representative mass spectra of the major identified metabolites are presented in Figures 8, 9, providing additional support for the proposed compound assignments.

The resulting chemical profiles revealed marked differences between extracts according to solvent polarity. As summarized in Table 5, a total of fifteen metabolites were detected in the hexane extract, while a smaller subset of metabolites was identified in the acetone fraction, reflecting differences in extraction selectivity associated with solvent polarity. The corresponding chromatographic profiles and representative mass spectra further confirmed the distinct chemical composition of each extract.

**TABLE 5:** Chemical profile of the hexane and acetone extracts of *Eryngium heterophyllum* by Gas Chromatography/Mass Spectrometry Analysis.

Sample	No.	Compound	*RT	Area (%)	Match (%)
Hexane extract	1	Tiglic acid	11.84	1.255	86.64
	2	1,2,4-trimethylbenzene	13.3	0.545	94.99
	3	2,5-dimethylbenzaldehyde	15.89	0.987	94.24
	4	2,4,5-trimethylbenzaldehyde	18.51	0.923	93.19
	5	2,4,5-trimethylbenzoic acid	19.49	20.748	90.75
	6	Caryophyllene oxide	19.92	1.269	94.42
	7	3-Buten-2-one, 4-(5,5-dimethyl-1-oxaspiro [2.5] oct-4-yl)	20.2	2.236	86.19
	8	Corymbolone	22.88	8.040	83.1
	9	Hexahydrofarnesylacetone	23.3	10.638	89.48
	10	Methyl palmitate	23.71	4.889	95.57
	11	Palmitic acid	23.81	1.660	95.74
	12	Ethyl palmitate	25.26	0.555	96.00
	13	Methyl stearate	29.15	1.520	85.94
	14	Nonacosane	32.78	9.335	84.8
	15	Tetratriacontane	34.42	14.254	82.61
Acetone extract	16	Methyl propenyl ketone	4.9	4.127	80.50
	1	Tiglic acid	6.04	3.661	83.81
	5	2,4,5-trimethylbenzaldehyde	14.88	16.061	97.05
	4	2,4,5-trimethylbenzoic acid	17.12	12.272	90.76
	10	Methyl palmitate	22.88	4.776	87.17
	11	Palmitic acid	23.29	25.362	94.71

*RT, retention time.

The hexane extract was predominantly composed of nonpolar metabolites, including long-chain hydrocarbons (e.g., nonacosane and tetratriacontane), fatty acids (palmitic acid), and their corresponding esters (methyl palmitate and ethyl palmitate), as well as sesquiterpene derivatives such as caryophyllene oxide. Notably, 2,4,5-trimethylbenzoic acid and hexahydrofarnesyl acetone were among the most abundant metabolites based on relative peak areas. In contrast, the acetone extract showed a distinct profile characterized by a higher relative abundance of aromatic metabolites, particularly 2,4,5-trimethylbenzaldehyde and 2,4,5-trimethylbenzoic acid, along with methyl ketones and fatty acid derivatives such as palmitic acid. These differences highlight the enrichment of moderately polar metabolites in the acetone fraction.

**FIGURE 10:**
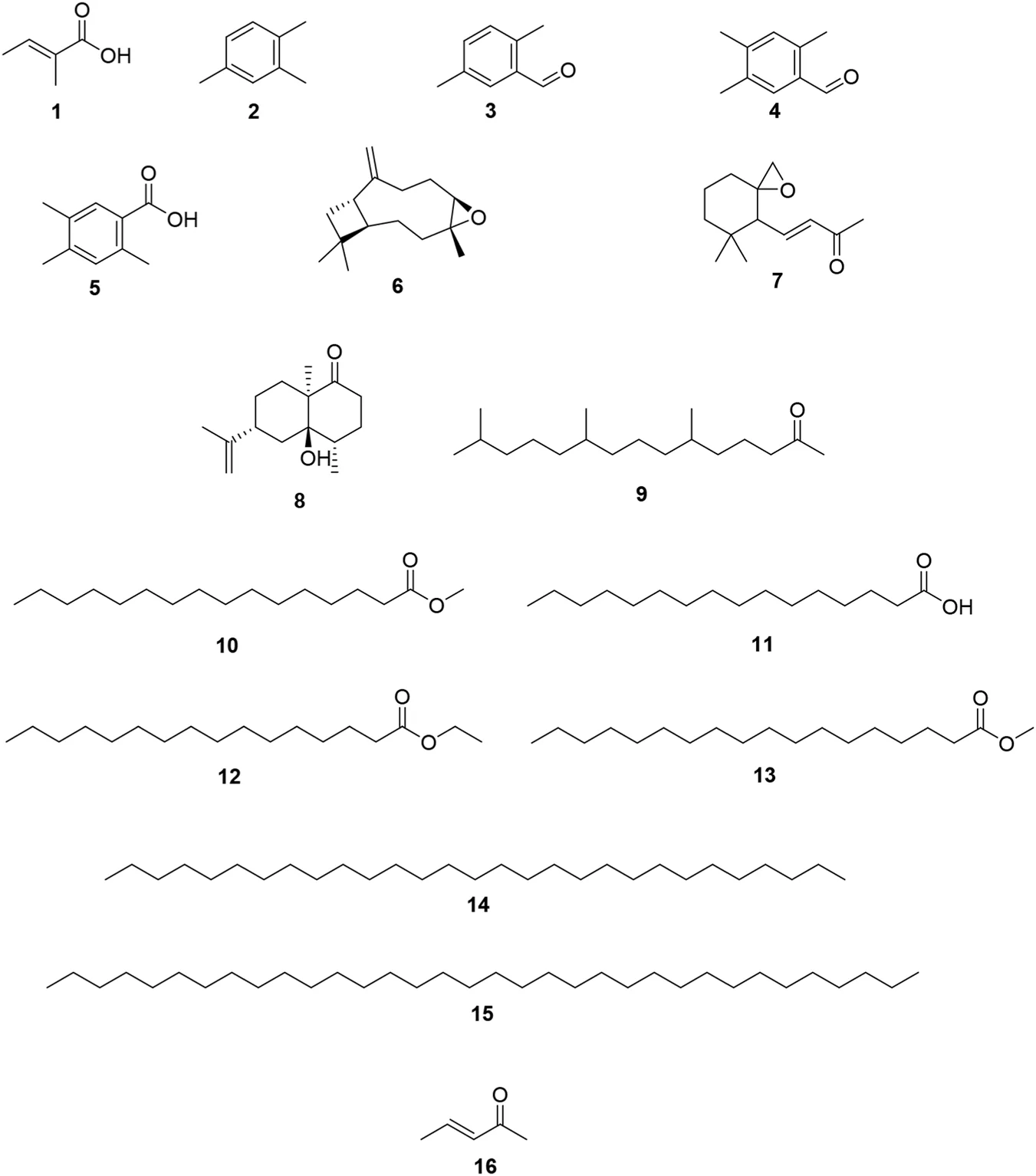
Chemical structures of metabolites 1-16 identified in the hexane and acetone extract of *Eryngium heterophyllum*. Structures were drawn using ChemDraw software (v.20.1.1) based on metabolites identified by GC-MS analysis.

To facilitate the visualization of the main metabolites identified in both extracts, the chemical structures of the most representative metabolites are presented in Figure 10. This figure includes major metabolites selected based on their relative abundance and potential biological relevance, allowing a clearer interpretation of the structural diversity associated with each fraction.

It should be noted that GC–MS analysis is primarily employed for the detection of volatile and semivolatile phytochemicals, including fatty acids, sterols, terpenoids, lipophilic metabolites, and some thermally stable metabolites (Al-Rubaye et al., 2017). In addition, previous studies have successfully applied GC–MS analysis to methanolic and polar plant extracts containing volatile or semivolatile metabolites (Gomathi et al., 2015; Choudhary et al., 2019; Mamza et al., 2012; Prakash, 2023). However, highly polar, high molecular weight, glycosylated, or thermolabile metabolites potentially present in the hydroalcoholic extract, including complex phenolics and glycosylated flavonoids, may not have been adequately detected under the experimental conditions employed in the present study. Therefore, future studies using complementary analytical approaches such as LC–MS/MS-based metabolomics will be important to achieve a more comprehensive phytochemical characterization of the hydroalcoholic fraction.

## 4 Discussion

Chemotherapy resistance represents a major clinical challenge that limits the long-term efficacy of anticancer drugs such as PTX. Although resistance is typically associated with advanced or refractory tumors, it is widely recognized that many of the underlying mechanisms—such as drug efflux, evasion of apoptosis, and activation of survival pathways—may already be present even in drug-sensitive cancer cells, thereby contributing to suboptimal therapeutic responses.

In this context, chemosensitization has emerged as a promising strategy to improve the efficacy of conventional chemotherapy (Kar et al., 2024; Eslami et al., 2024). Chemosensitizing agents, particularly those derived from natural products, can enhance drug response by modulating key cellular pathways involved in survival and stress adaptation, even in the absence of established resistance (Kumar and Jaitak, 2019; Zou et al., 2024).

These metabolites include various MDR-modulating molecules previously reported in natural products, such as flavonoids, lactones, chalcones, alkaloids, curcuminoids, and catechins, which have been associated with the modulation of drug transport, apoptosis-associated pathways, and chemotherapy response, thereby potentiating the cytotoxic effects of chemotherapeutic agents (Sánchez-Carranza et al., 2024; Dewanjee et al., 2017). In line with this approach, the present study demonstrates that extracts from *Eryngium heterophyllum* not only exhibit intrinsic cytotoxic activity in A549 lung cancer cells but also significantly enhance the cytotoxicity of PTX.

It is important to note that the chemosensitizing effect observed in the present study is consistent with previous reports describing synergistic interactions between *Eryngium*-derived extracts and conventional chemotherapeutic agents. Karaboğa Arslan et al. demonstrated that *Eryngium kotschyi* extracts potentiated the growth-inhibitory effects of cisplatin and doxorubicin in RL95-2 endometrial cancer cells, supporting the idea that species of the genus *Eryngium* may contain bioactive metabolites capable of potentiating chemotherapy (Karaboğa Arslan et al., 2022). Although the tumor model and chemotherapeutic agents differ from those used in this study, both suggest that *Eryngium* extracts can act not only as direct cytotoxic agents but also as modulators of the chemotherapy response.

Although previous studies have described antiproliferative and antioxidant properties of *Eryngium spe*cies, most reports have primarily focused on their intrinsic cytotoxic or ethnopharmacological activities. Previous studies in *E. heterophyllum* have reported phenolic metabolites and phytosterols such as rutin, ferulic acid, caffeic acid, β-sitosterol, stigmasterol, and falcarindiol, which have been associated with cytotoxic, antioxidant, and apoptosis-related activities in different experimental models (Villa-Santiago et al., 2025; Galván-Ciprés et al., 2026). These observations may support the possible contribution of multiple phytochemical groups to the biological effects observed in the present study. In contrast, the present study goes beyond the conventional evaluation of direct cytotoxicity by demonstrating, for the first time, the chemosensitizing capacity of *E. heterophyllum* extracts in combination with paclitaxel in lung cancer cells. Importantly, our findings reveal not only synergistic interactions with PTX, but also functional modulation of multidrug resistance-associated mechanisms, including increased intracellular doxorubicin retention and potential interference with P-gp mediated efflux. These observations distinguish the present work from previous reports and position *E. heterophyllum* as a promising source of adjuvant agents capable of enhancing chemotherapy efficacy.

These findings highlight their potential as adjuvant agents capable of improving chemotherapy efficacy and suggest their possible relevance in delaying or counteracting the onset of drug resistance.

Among the evaluated fractions, the hexane extract exhibited the highest intrinsic cytotoxic potency (IC_50_ ≈ 25 μg/mL), suggesting that low-polarity metabolites play a major role in direct antitumor activity. This observation is consistent with previous phytochemical and pharmacological studies on the genus *Eryngium*, in which nonpolar fractions have consistently demonstrated enhanced cytotoxic and antiproliferative effects. Such activity has been largely attributed to the presence of triterpenes, sterols, and other lipophilic metabolites capable of disrupting membrane integrity, modulating mitochondrial function, and inducing apoptosis (Cárdenas-Valdovinos et al., 2023; Villa-Santiago et al., 2025).

In particular, published studies have demonstrated that extracts from *Eryngium species* exhibit significant cytotoxic activity against cancer cell lines, including the induction of apoptosis and cell cycle arrest (Castañeda Espinoza et al., 2026; Vukic et al., 2018). More recent research has further characterized the antiproliferative properties of *Eryngium*-derived extracts, linking their bioactivity to phenolic composition and redox modulation capacity. Similarly, ethnopharmacological studies have provided additional evidence supporting the anticancer potential of this genus, including the inhibition of tumor cell proliferation and the activation of apoptosis-related pathways (Yurdakök and Baydan, 2013; Hermosaningtyas et al., 2024).

However, specific evidence regarding the antiproliferative activity of *E. heterophyllum* remains limited, underscoring the relevance of the present findings.

In the present study, a clear potentiating effect on PTX activity was observed. However, when the chemosensitizing capacity was examined, the acetone extract produced the most pronounced synergistic interaction (CI = 0.35), followed by the hexane (CI = 0.47) and hydroalcoholic extracts (CI = 0.58). This divergence between intrinsic cytotoxicity and chemosensitizing potential is mechanistically relevant and suggests that distinct classes of phytochemicals are involved in each effect.

The synergistic interaction observed between *E. heterophyllum* extracts and paclitaxel is consistent with previous reports demonstrating that plant-derived metabolites can enhance paclitaxel sensitivity through the modulation of apoptosis-related pathways and multidrug resistance mechanisms. For example, Sanchez-Carranza et al. reported that achillin increased paclitaxel sensitivity in resistant Hep3B/PTX cells, promoting apoptosis and overcoming drug resistance. Similarly, Zhou et al. demonstrated that curcumin potentiated PTX activity through the modulation of NF-κB-associated signaling pathways. Furthermore, a recent systematic review by Asnaashari et al. highlighted the ability of flavonoids and other phytochemicals to synergize with paclitaxel across multiple cancer models, supporting the use of natural products as chemosensitizing agents.

These observations suggest that while lipophilic metabolites may contribute primarily to direct cytotoxicity, moderately polar metabolites—likely enriched in the acetone fraction—could participate in the modulation of drug response pathways. Notably, the stronger synergistic effect observed for the acetone extract is in agreement with previous reports indicating that flavonoid-rich fractions are frequently associated with enhanced chemotherapy response through the regulation of apoptosis, oxidative stress, and multidrug resistance-associated pathways. Similar effects have been reported for natural lipophilic and polyphenolic metabolites, which have demonstrated the ability to enhance chemotherapy response and modulate mechanisms associated with oxidative stress, apoptosis, and multidrug resistance (Carrasco-Torres et al., 2017; Rarison et al., 2023; Oršolić and Jazvinšćak Jembrek, 2024).

The present study extends our previous findings on the cytotoxic activity of *E. heterophyllum* extracts (Castañeda-Espinoza et al., 2026), by exploring their potential chemosensitizing properties. While earlier studies demonstrated selective cytotoxicity toward cancer cells, the current work provides evidence that these extracts may enhance the antiproliferative effect of paclitaxel and increase intracellular doxorubicin retention, suggesting a possible modulation of drug transport-related mechanisms.

This interpretation is supported by previous studies performed in A549 cells, where intracellular accumulation of doxorubicin was shown to be influenced by P-glycoprotein activity. Rogalska et al. demonstrated that inhibition of P-gp with verapamil significantly increased intracellular doxorubicin accumulation in A549 cells, supporting the use of this cell line for evaluating the modulation of drug efflux mechanisms and anthracycline retention. The rightward fluorescence shift observed in the present study follows a similar pattern, suggesting that the extracts may interfere with transport processes involved in drug extrusion (Rogalska et al., 2014).

These findings broaden the pharmacological relevance of this species beyond its direct cytotoxic activity.

For this part, the phytochemical characterization of *E. heterophyllum* by GC–MS revealed a profile of secondary metabolites that complements and extends the knowledge reported for the genus in the central-western region of México (Cárdenas-Valdovinos et al., 2023). While the literature highlights the prevalence of phenolic acids (rosmarinic, chlorogenic, and caffeic acids) and flavonoids (quercetin and kaempferol) as the main polar metabolites of the genus *Eryngium*, our results demonstrate that the nonpolar and moderately polar fractions of *E. heterophyllum* exhibit a chemical composition dominated by long-chain hydrocarbons, fatty acids, and specific aromatic aldehydes.

The hexane extract showed a significant abundance of nonpolar metabolites, particularly nonacosane (9.33%) and tetratriacontane (14.25%), along with palmitic acid and its ester derivatives. The presence of palmitic acid is consistent with reports for other species of the genus, such as *Eryngium carlinae*; however, the high concentration of long-chain hydrocarbons in this fraction suggests a biological mechanism of action associated with membrane disruption, mitochondrial dysfunction, and induction of apoptosis (Wang et al., 2023; Al Kamaly et al., 2024). These findings provide a mechanistic basis for the intrinsic cytotoxicity observed in this fraction, which is particularly relevant given that previous studies on *E. heterophyllum* have primarily focused on its hypocholesterolemic and trypanocidal effects.

On the other hand, the acetone extract showed an enrichment in moderately polar aromatic metabolites, notably 2,4,5-trimethylbenzaldehyde (16.06%) and 2,4,5-trimethylbenzoic acid (12.27%). These metabolites possess structural features that enable their participation in redox processes and the modulation of intracellular signaling pathways. This chemical composition may explain the superior chemosensitizing capacity of the acetone extract compared to the hexane extract, suggesting that these metabolites could interfere with drug resistance mechanisms through the regulation of oxidative stress and the potential modulation of efflux transporters (Dell’Olmo et al., 2021).

Notably, caryophyllene oxide, although in low concentration, was present in the hexane extract. This sesquiterpene has been widely documented for its pro-apoptotic and chemosensitizing effects in the literature, supporting the hypothesis that the biological activity of *E. heterophyllum* does not rely on a single compound but rather on the synergistic interaction of multiple molecular targets (Fidyt et al., 2016).

In contrast to species such as *E. carlinae*, which has been extensively studied for its antioxidant and renoprotective properties associated with phenolic metabolites (Trejo-Hurtado et al., 2023; Pérez-Muñoz et al., 2022). Our findings for *E. heterophyllum* highlight the potential of its less polar fractions as sources of chemotherapeutic and chemosensitizing agents. The coexistence of lipophilic cytotoxic metabolites and moderately polar redox-active metabolites supports the use of fractionation strategies to unravel the complex pharmacological behavior of plant extracts. The integration of these chemical profiles with the ethnobotanical knowledge reported by Cárdenas-Valdovinos et al. underscores the relevance of *E. heterophyllum* as a promising source of bioactive metabolites with synergistic therapeutic applications, thereby providing scientific validation for its role in traditional Mexican medicine (Cárdenas-Valdovinos et al., 2023).

The hydroalcoholic extract showed the highest total phenol content, while the acetone extract exhibited the highest concentration of flavonoids. These differences are consistent with the polarity of the extraction solvents and suggest selective enrichment of different phytochemical groups. Phenolic metabolites and flavonoids have been widely associated with antioxidant, cytotoxic, and chemosensitizing activities due to their ability to modulate oxidative stress, apoptosis, and pathways associated with multidrug resistance. Interestingly, although the hydroalcoholic extract had a higher total phenol content, the acetone extract showed superior chemosensitizing activity, suggesting that the observed biological effects may depend not only on the total amount of phenols but also on the specific composition of bioactive metabolites present in each fraction.

Taken together, the chemical composition revealed by GC-MS analysis provides a plausible mechanistic basis for the dual activity of *E. heterophyllum* extracts, linking specific classes of metabolites to either direct cytotoxic effects or modulation of drug response.

Another strength of the study is the observed selectivity toward tumor cells, particularly for the acetone extract (SI = 8.2), suggesting a potentially favorable therapeutic window. This characteristic may be related, at least in part, to the redox-modulating properties of the phytochemicals present in the extracts. It is increasingly recognized that plant-derived metabolites can exert both antioxidant and pro-oxidant effects depending on the cellular context and redox status. In cancer cells, which typically exhibit elevated basal ROS levels, certain phytochemicals may further disrupt redox homeostasis, thereby promoting oxidative stress-associated cell death, while exerting less pronounced effects on non-tumor cells (Sun et al., 2024). Therefore, the cytotoxic and chemosensitizing activities observed in the present study may be associated with complex redox-related mechanisms rather than exclusively antioxidant effects. However, the involvement of ROS modulation was not directly evaluated and warrants further investigation. (Petronek et al., 2021; Ranbhise et al., 2025).

Although previous studies have described antiproliferative and antioxidant properties of *E. species*, most reports have primarily focused on their intrinsic cytotoxic or ethnopharmacological activities. In contrast, the present study goes beyond the conventional evaluation of direct cytotoxicity by demonstrating, for the first time, the chemosensitizing capacity of *E. heterophyllum* extracts in combination with PTX in lung cancer cells. Importantly, our findings reveal not only synergistic interactions with PTX, but also functional modulation of multidrug resistance-associated mechanisms, including increased intracellular doxorubicin retention and potential interference with P-gp-mediated efflux. These observations distinguish the present work from previous reports and position *E. heterophyllum* as a promising source of adjuvant agents capable of enhancing chemotherapy efficacy.

Future studies should focus on identifying the specific bioactive metabolites responsible for the observed chemosensitizing effects and validating their activity in drug-resistant lung cancer models and *in vivo* systems. In addition, further mechanistic studies are warranted to determine the involvement of molecular pathways associated with multidrug resistance, apoptosis, oxidative stress, and survival signaling. Biomarkers such as ABCB1/P-gp, MRP1, BCRP, BAX, BCL-2, caspase-3, PI3K/AKT, NF-κB, and ROS-related pathways could provide deeper insight into the mechanisms underlying the synergistic interaction between *E. heterophyllum* extracts and PTX. These perspectives are supported by recent evidence demonstrating that natural metabolites can act as chemosensitizing agents through the simultaneous modulation of multidrug resistance transporters, apoptotic pathways, oxidative stress responses, and pro-survival signaling networks (Sánchez-Carranza et al., 2024; Bharathiraja et al., 2023; Dinić et al., 2018). Such multitarget mechanisms have emerged as a promising strategy for overcoming chemotherapy resistance and may explain the superior chemosensitizing activity observed for the acetone extract in the present study. Accordingly, further elucidation of these molecular targets may contribute to the development of novel natural-product-based adjuvant therapies for lung cancer treatment.

To our knowledge, this is the first study demonstrating the chemosensitizing activity of *E. heterophyllum* extracts in combination with PTX through modulation of multidrug resistance-associated mechanisms in lung cancer cells, positioning this species as a promising source of multi-target chemosensitizing agents and supporting research into its bioactive metabolites in combination therapies against lung cancer.

## 5 Conclusion

The present study demonstrates that *E. heterophyllum* extracts possess both cytotoxic and chemosensitizing activities in A549 lung cancer cells. Among the evaluated fractions, the acetone extract exhibited the strongest chemosensitizing effect in combination with PTX, while the hexane extract showed the highest intrinsic cytotoxic activity. Furthermore, the extracts promoted intracellular doxorubicin retention and modulated P-gp expression, suggesting a potential effect on multidrug resistance-associated mechanisms. Although further studies are required to identify the active metabolites and validate the underlying molecular pathways, these findings support *E. heterophyllum* as a promising source of natural metabolites with potential applications as adjuvants in lung cancer chemotherapy.

## Data Availability

The raw data supporting the conclusions of this article will be made available by the authors, without undue reservation.
